# Semantic concept schema of the linear mixed model of experimental observations

**DOI:** 10.1038/s41597-020-0409-7

**Published:** 2020-02-27

**Authors:** Hanna Ćwiek-Kupczyńska, Katarzyna Filipiak, Augustyn Markiewicz, Philippe Rocca-Serra, Alejandra N. Gonzalez-Beltran, Susanna-Assunta Sansone, Emilie J. Millet, Fred van Eeuwijk, Agnieszka Ławrynowicz, Paweł Krajewski

**Affiliations:** 1 Institute of Plant Genetics, Polish Academy of Sciences, ul. Strzeszyńska 34, 60-479 Poznań, Poland; 2 Institute of Mathematics, Poznań University of Technology, ul. Piotrowo 3a, 60-965 Poznań, Poland; 3 Department of Mathematical and Statistical Methods, Poznań University of Life Sciences, ul. Wojska Polskiego 28, 60-637 Poznań, Poland; 4 Oxford e-Research Center, Department of Engineering Science, University of Oxford, Oxford, OX1 3QG UK; 5 Biometris, Wageningen University & Research Centre, P.O. Box 16, 6700 AA Wageningen, The Netherlands; 6 Faculty of Computing and Telecommunications, Poznan University of Technology, ul. Piotrowo 3, 60-965 Poznań, Poland; 7 Present Address: Scientific Computing Department, Science and Technology Facilities Council, UK Research & Innovation, Didcot, OX11 0QX UK

**Keywords:** Computational models, Statistical methods, Data processing, Classification and taxonomy, Data integration

## Abstract

In the information age, smart data modelling and data management can be carried out to address the wealth of data produced in scientific experiments. In this paper, we propose a semantic model for the statistical analysis of datasets by linear mixed models. We tie together disparate statistical concepts in an interdisciplinary context through the application of ontologies, in particular the Statistics Ontology (STATO), to produce FAIR data summaries. We hope to improve the general understanding of statistical modelling and thus contribute to a better description of the statistical conclusions from data analysis, allowing their efficient exploration and automated processing.

## Introduction

The amount of scientific data is rapidly increasing; hence, efficient approaches for processing and managing the data are required. The sharing of comprehensive data summaries that include adequate metadata is a way to knowingly address the data flood and to enable dataset discoverability and reuse. In the case of experimental data, information regarding the analytical objectives of the underlying scientific experiments and their statistical conclusions can advance the search for, preselection of and comprehension of the datasets by other researchers.

Although the publication of raw scientific data is generally encouraged, there is still unexploited potential in sharing and reusing derived statistical information about scientific datasets. Typically, the conclusions from statistical analysis are not being published in any universal, computer-enabled form that allows automated processing. We demonstrate the idea of sharing the statistical information of datasets in a FAIR (Findable, Accessible, Interoperable and Reusable)^1^ way for the purpose of analysis documentation and data exploration, exemplified by statistical modelling.

In the context of scientific experiments following a factorial design, in which quantitative variables are observed, one of the statistical methods frequently used to process experimental data is linear mixed model (LMM) fitting^2^. The analysis executed with the use of the LMM is, in many cases, sufficient for statistical inference, as it allows us to examine the role and significance of the experimental factors and draw statistical conclusions about the variables of interest. Data analysis by the LMM approach is a way to uniformly process datasets from designed experiments so that the obtained parameters are interpretable and comparable. The modelling outcomes constitute an informative summary of the experimental results and can be used as criteria for data discovery.

Most precise information about a statistical model is conveyed by the algebraic formulations and - for the practical purposes of computation - by means of statistical programming languages used in packages such as R, SAS or Genstat. They convey direct, complete information about the model specification and allow one to access all parts of the analytical procedure and its results, as well as to rerun the analysis, provided the same computational environment is available (cf.^3^). However, different approaches and terminologies for specifying the model within scripts, as well as various output formats from the statistical packages, make it difficult to share the model description directly between the tools and their users and to deliver the analysis conclusions to researchers. Hence, there is a need for a more convenient and interoperable way to expose the analysis details.

A natural language description is typically used in papers to summarise the research results and methodology. The meaning of the “concepts” conveyed in scientific publications has been traditionally based on common understanding or by referring to other publications in the field or to general dictionaries or glossaries. In the case of statistical terms, the traditional references include resources such as the OECD Dictionary of Statistical Terms (https://stats.oecd.org/glossary), Encyclopedia of Statistical Sciences^4^, or The Oxford Dictionary of Statistical Terms^5^. While more accessible by a wide range of users, natural language descriptions frequently lack precision, preclude computer readability and further automated processing.

Semantic web technologies^6^ provide a way to represent data together with their semantics that are comprehensible for both human agents and computer processing. The formalisation of knowledge is done according to the Resource Description Framework (RDF), where facts comprising a data model are represented as triples that together form a knowledge graph. Graphs that attempt to encompass the entities from a particular domain, together with all their properties (e.g., definitions) and relations, are called ontologies; they can be represented in the Web Ontology Language (OWL). The nodes in the graph represent the entities (pieces of information) and the links between nodes - their relationships. All resources (nodes and links) are identified by Internationalised Resource Identifiers (IRIs) - persistent and resolvable names that enable interactions with their representations. By describing data through references to ontology resources that formalise common knowledge, explicit definitions can be provided and later utilised to understand the structured data. Thus, use of a semantic data model is a flexible, precise and interoperable way to describe the methodology and the results of experimental data processing in the context of statistical theory and broader schema of knowledge. Semantic approaches have been implemented to expose many types of data resources on the web, which contribute to the network of Linked Data^7^. Knowledge-based systems are used for data management in specific areas of biomedical research^8,9^. For the semantic representation of multidimensional data and their aggregations, a Data Cube vocabulary (https://www.w3.org/TR/vocab-data-cube) is broadly used to annotate the computed statistics. For a more detailed description of the custom experiment and analysis models, the statistics ontology (STATO) (http://stato-ontology.org) can be used (as in, e.g., a neuroimaging data model: http://nidm.nidash.org/specs/nidm-results).

In this paper, we propose a semantic model for the results (or derived data) obtained from LMM analysis. Our hypothesis is that a structured model of the analysis can advance the exploration of experimental datasets by enabling automated processing of the modelling results. Moreover, a semantic model of the LMM, which provides a comprehensive view of the data analysis and its results, can bridge the gap between statisticians, developers, data managers, and researchers and improve their mutual understanding of the experimental data and scientific conclusions. Thus, we assume that it can contribute to enhanced data interoperability and support reproducible research.

The main goal of our work is to accurately model the statistical field of the LMM with existing ontologies (extending them where necessary) to provide a comprehensive description of the analysis process and its results. The main contribution of our work is the structural description of the LMM analysis, i.e., the identification of its key statistical concepts and the selection of ontological terms with which to annotate them. By formulating a list of competency questions about linear mixed modelling for the ontology, we pinpoint the gaps in the existing ontologies and propose additions to them that can fill in those gaps. We demonstrate the use of the developed solutions for describing the LMM analysis (univariate and multivariate) to facilitate experimental data publication and management. We concentrate on LMMs with binary design matrices, with the possible addition of continuous covariates and with one error term with homogeneous variance, which form a simple subclass of all LMMs and are frequently used to analyse the research datasets from designed experiments. We demonstrate the application of semantic models for the identification of LMM analyses of scientific experiments on plants by providing sample query cases that explain the motivations and benefits for domain researchers.

## Methods

### Motivation

**Fig. 1:**
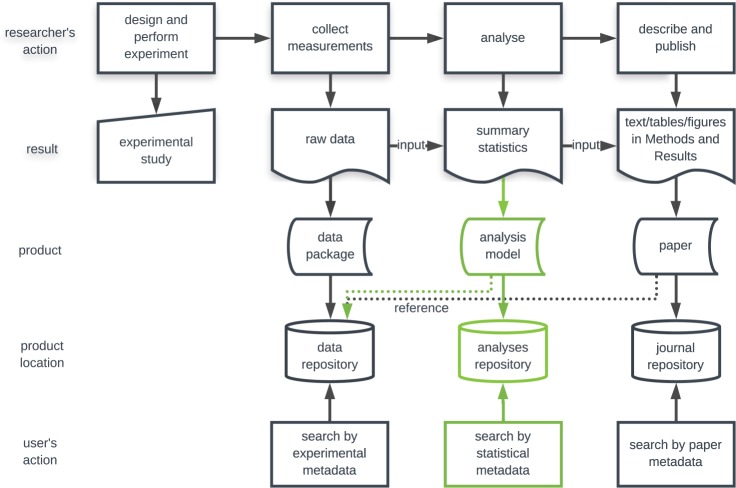
Position of statistical analysis in the life cycle of scientific data. The green path corresponds to the contribution of our work to scientific data discovery. By storing computer-readable summaries of statistical analyses, the search for data according to the corresponding statistical conclusions is enabled, as well as the potential aggregation of results.

Currently, data management policies increasingly support open science^10^. They stimulate the sharing of scientific data in an accessible and interoperable way and emphasise the need to include adequate experimental metadata^11^ to make the data findable and reusable. However, the focus is usually on raw data. Little attention is paid to the publication of statistically sound, well-documented and computationally enabled data summaries. Conclusions from scientific experiments obtained from data analyses are being published in papers mainly by means of textual descriptions, tables and figures, which do not allow for their automated processing. As a result, public datasets in searchable repositories can usually only be discovered by experimental metadata and not by conclusions from their analysis. Our approach aspires to document the statistical analysis of a dataset in a FAIR way, which enables the analytical results (such as statistical tests or summary statistics) to be suitable for automated processing and fills in the gaps in scientific data publishing (Fig. 1).

### Linear mixed modelling

Linear mixed modelling is a flexible approach that can be used to analyse data where some observed variables are explained by means of other coexisting variables. Different types of LMMs and the corresponding estimation methods are used in applied sciences. Here, we consider typical use cases of LMMs for analyses of the results of scientific experiments where a trait of interest (e.g., crop yield or body weight) observed for a number of experimental units (samples or individuals, e.g., plants or animals) is investigated in the context of several experimental factors (e.g., species, watering or dietary regimes) or other observed traits (e.g., plant height or animal age). The goal is to infer the influence of particular characteristics on the trait of interest (e.g., answering questions such as “Does crop yield depend on the variety?” or “What are the effects of particular treatments on body weight?”).

From a mathematical point of view, LMM analysis aims at quantifying the relationship between dependent variables and a set of independent variables through a parametrised linear function. Depending on the number of variables modelled simultaneously, univariate and multivariate models can be considered. Model parameters represent effects whose estimates and significance are typically of interest. Two types of effects are inherent in LMMs: fixed and random. Fixed effects are used to model the effects of individual levels of a categorical variable or of a continuous variable. Random effects express additional model variability, which is usually represented by parametrised covariance structures chosen according to the assumed properties of the corresponding categorical variables. The remaining variability, not included otherwise in the model, is represented by a presumed parametrised error covariance structure. The overall quality of the fitted model (i.e., its adequacy to the data) is assessed by investigating the output of the estimation and significance tests and the model quality (goodness of fit) criteria.

A detailed mathematical explanation of the LMM and its analysis, which provides a theoretical background for the semantic model proposed below, can be found in Supplementary File 1 and the previous literature cited there^12–28^.

### Semantic modelling

To formalise the above description of the LMMs, we make use of the terms defined in publicly available vocabularies. We intend to provide universal definitions of the LMM-related concepts, available both to human readers and to be read automatically by computers. Additionally, by referring to public terms, we aim at embedding the concepts related to the LMM in a more general knowledge schema, with the goal of making data interoperable and interlinked within the Linked Data cloud^7^. Thus, through a semantic model of statistical analysis, expressed as an RDF graph published in a triple store, a set of statistical assumptions and conclusions becomes FAIR: findable (identifiable and searchable), accessible (retrievable via a standard protocol), interoperable (described with formal language, with reference to the original data), and re-usable (equipped with accurate and relevant attributes).

There exist a few terminologies that address statistical terms. The STATO is a general ontology that aggregates rich statistical vocabulary with its textual and formal definitions and allows its classification. It relies on an upper-level ontology (the Basic Formal Ontology) frequently used in many fields of research, notably in the life sciences. It is also compatible with other ontologies that are part of the Open Biological and Biomedical Ontology (OBO) Foundry. Thus, by taking advantage of the general framework of the STATO to annotate the LMM entities, we can put the modelled knowledge in the broad interdisciplinary context.

The proposed model aims to provide sufficient semantics for the elements of the LMM to be used by experimental data managers and domain experts to study the assumptions and conclusions from the statistical analysis of datasets. However, it is not supposed to capture all computational or statistical technicalities. Our approach aims at an accurate compromise between the theoretical profoundness and usability of the solution for the needs of experimental data management and dataset discovery. If needed, it should be possible to extend our approach with a more detailed and accurate projection of some concepts (e.g., by further partitioning some processes into basic statistical operations and their components).

**Fig. 2:**
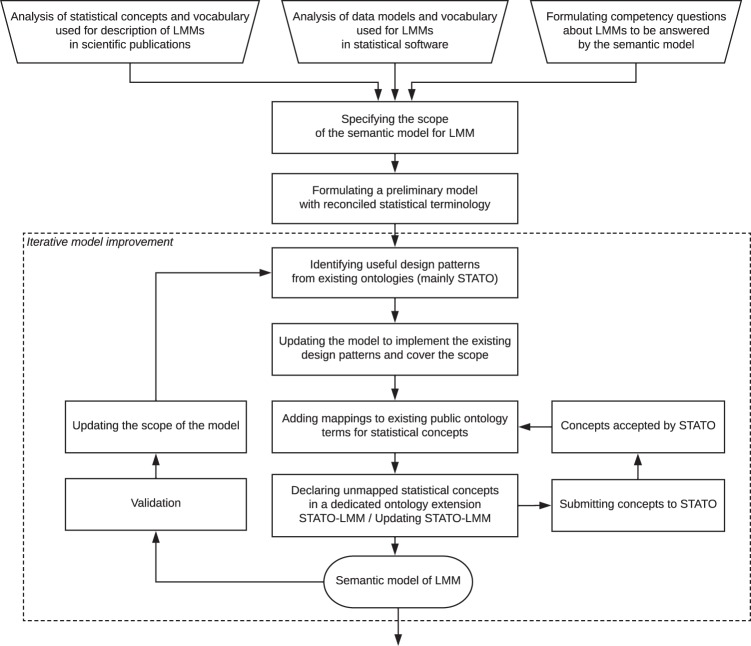
Workflow of the development of the semantic model for the LMM. After formulating the requirements and scope of the model, an iterative procedure was used to improve the model by incorporating existing design patterns and concepts from public ontologies (mainly from the STATO) and requesting the addition of new terms to public resources (STATO). An ontology extension STATO-LMM is maintained to accommodate terms not present in other public resources.

**Fig. 3:**
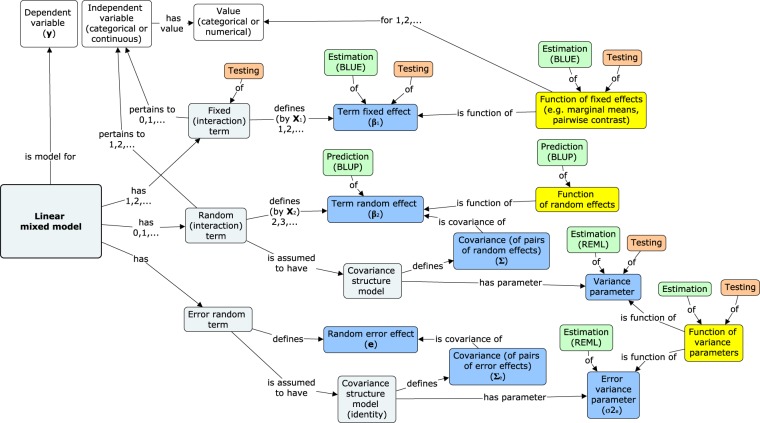
Graphical representation of the main concepts pertaining to a linear mixed model. Nodes, marked with symbols as in formula (1), correspond to model components. Edges with the informal notation “0, 1, …” represent the character and cardinality of the relationships. The node labelled as *linear mixed model* represents the LMM itself. It is linked directly to the *dependent variable* and indirectly, through model *terms*, to *independent variables*. Model *terms* define *effects* according to the *design matrices*. *Random terms* are linked to their *covariance structure models*, which define *covariances* of the *random effect*, and are characterised by *variance parameters*. All model parameters [dark blue nodes] can have their *estimates* (accompanied by their *standard errors* and methods) [green], and all (except *random effects*) can be the subject of *hypotheses* (with *test statistic values*, *degrees of freedom*, *p-values*, or other uncertainty indicators) [orange]. *Parametric functions* [yellow] can be defined on all model parameters. *Variables* [white] are given in an external dataset.

The process of constructing a semantic model of LMM analysis is illustrated in Fig. 2. To specify the initial scope and requirements of the semantic model, we have summarised the concepts used in publications from the field of mathematical statistics and formulated a number of questions about the cases of LMM analyses that should be answered by the model. To formally rephrase the queries, the statistical concepts were linked to classes and properties defined in the ontology according to the following steps:

1. prepare a preliminary, informal model of statistical concepts related to the LMM (Fig. 3),

2. find direct representations of the basic LMM concepts by appropriate classes in the STATO; analyse existing ontology design patterns and properties of the selected classes to identify additional entities useful for describing the LMM (and not included in the initial model specification); and adjust the model to match the existing ontological classes and relations,

3. specify concepts missing from the ontologies in a local resource to extend the STATO, resulting in the STATO-LMM,

4. formalise the semantic model representation by mapping statistical concepts to ontology terms (62 entities reused from the STATO, 38 entities declared in the STATO-LMM),

5. request to extend the STATO with the missing concepts; continuously update the model whenever a term is added to the STATO with the new identifier (22 entities accepted at the point of preparing the manuscript),

6. validate the model against the requirements formulated through the initial questions, extend the scope where necessary and update the model with ontology terms accordingly.

A summary of the modelling process is given in Supplementary File 2. To facilitate access to the set of classes proposed for the semantic model of the LMM, an ontology module was extracted from the STATO (using the Ontofox tool^29^ which implements the MIREOT methodology^30^) and imported to an extended STATO-LMM ontology so that all the necessary terms could be easily found.

### Semantic model of the LMM analysis

Here, we describe how specific aspects of the statistical model fitting process can be semantically modelled with ontology terms (given below in italics), with their interdependence and involvement in different subprocesses of the model fitting taken into account. The figures illustrate the proposed semantics by depicting the class- and instance-level relationships. In later sections of the paper, we give complete examples of the models for the LMM analyses.

**Fig. 4:**
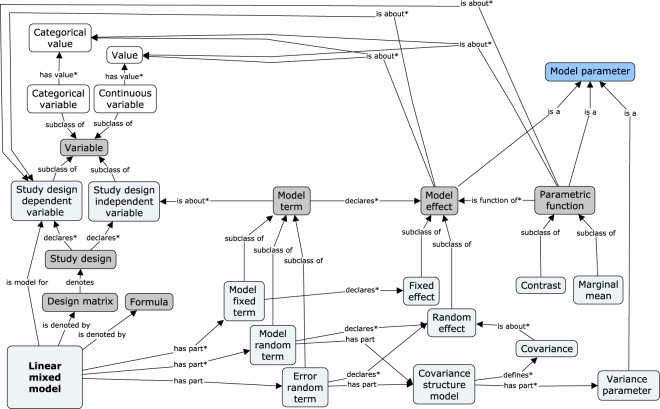
Concept schema for LMM declaration; semantic model of the core elements of the statistical model. Dark grey nodes represent concepts that were not explicitly present in Fig. 3. Asterisks (*) mark the relationships that can appear multiple times for a particular initial node.

First, we focus on the declaration of a linear mixed model (Fig. 4). The model itself is represented by an instance of the STATO *linear mixed model* class. It can be *denoted by* a *formula* to provide its corresponding mathematical or software-specific notation for simple human readability. The explicit semantics for the model structure can be declared through its components: terms and design matrix.

In our basic approach, which is driven by practical applications of the standard types of the models to the experimental datasets, a model is usually declared by specifying its *terms* (each one being *part of* the model), typically at least one fixed term, at least one random term, and one error term. A term is linked to its corresponding variables via the *is about* relationship, with one link per variable. An interaction term is modelled by using multiple links to variables, while the intercept term has none. This approach corresponds to declaring a model by a descriptive equation, as used in statistical software suites (e.g., *dependentVariable* = *independentVariable* + *error*).

The model can also be *denoted by* a *design matrix*. In this paper, we consider design matrices of the binary form, sufficient for LMMs usually used for designed experiments; thus, the term *design matrix* does not appear explicitly in Fig. 3, although we indicate its role in the definition of model effects. The *design matrix* allows for the consideration of more general designs and thus for the declaring and annotating of more model types than can be done through model terms. The *design matrix* class *denotes* the *study design* (which, by definition, encapsulates the specifications and protocols of the study at the origin of the modelled *dataset*), which *declares* the roles of the involved variables as *study design dependent* and *independent variables*. The former are always instances of the *continuous variable* class and are additionally linked to the model via the *is model for* relationship. *Independent variables* can be either *continuous* or *categorical variables*.

Quantitative measures of the model fit^31,32^ (such as the deviance, AIC, and BIC) can be added to the model as specific criteria classes from the STATO and linked to the model via a generic *is about* relationship.

Now, let us consider the semantics of the LMM effects and their derived concepts. Each effect is represented by a *model parameter* that is linked to the corresponding values of the independent and dependent variables via *is about* relationships. In the case of continuous covariates, the effect, being a regression coefficient, is attached to the value of 1 of the independent variables.

**Fig. 5:**
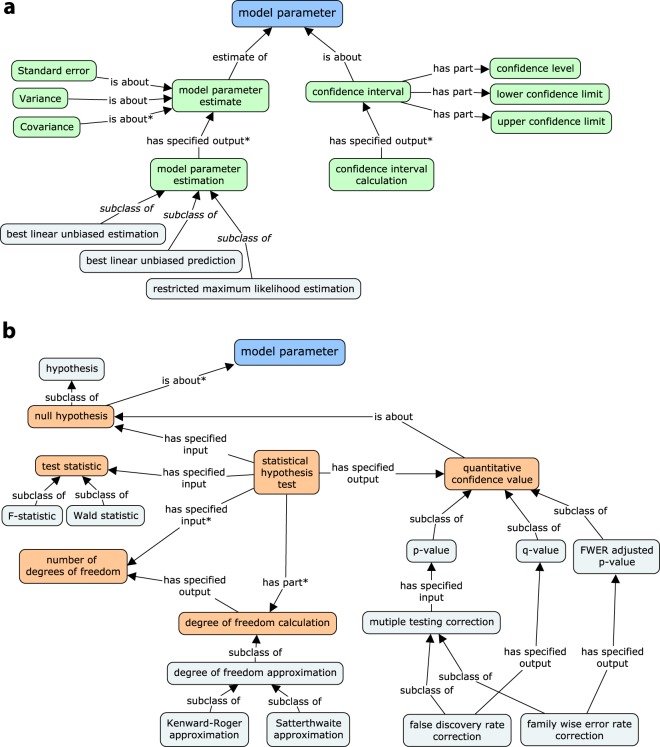
Concept schema for statistical inference in LMM analysis. (**a**) Model parameter estimation and confidence interval calculation. (**b**) Model parameter hypothesis testing.

All model effects and other statistical quantities that undergo estimation or testing procedures are considered instances of the *model parameter* class. A schema of concepts related to the *model parameter* class includes two main types of inferences about the model parameters, that is, estimation (Fig. 5a) and hypothesis testing (Fig. 5b). On the estimation side, both point *model parameter estimates* (together with their *standard errors* and *covariances*) and *confidence intervals* are represented as outputs of the adequate statistical processes. The *model parameter estimation* class encompasses all specialised estimation processes, such as variance parameter estimation, fixed effect estimation, and random effect prediction. On the testing side, the verification of a *null hypothesis* being *about* the *model parameter(s)* is achieved by a *statistical hypothesis test*, with the input formed by the *hypothesis*, *test statistic* and *number of degrees of freedom*. The output of the test, a *p-value*, which is possibly transformed into a *q-value* or according to a *family-wise error rate*, characterises the *null hypothesis* and enables *drawing a conclusion*.

**Fig. 6:**
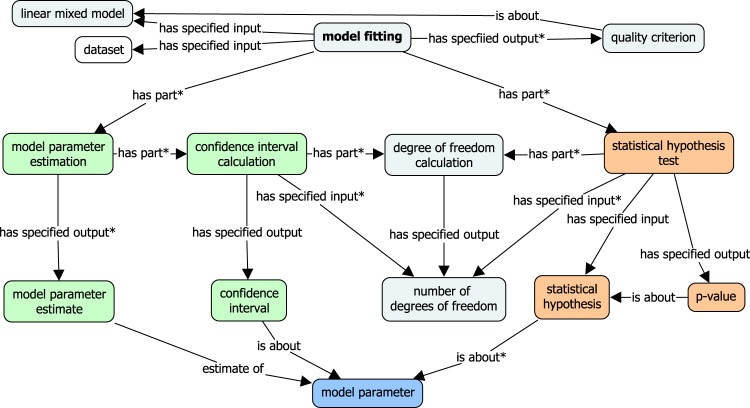
Concept schema of LMM execution: core processes involved in model fitting.

In Fig. 6, the model fitting procedure is presented from the processing perspective. The process of *model fitting* evaluates whether a *model* appropriately represents a *dataset* and assesses the *quality of fit* of the model. Primary processes constituting (via the *has part* relationship) the LMM analysis are *model parameter estimation*, *confidence interval calculation*, and *statistical hypothesis testing*. The main inputs and outputs of the operations are shown in the graph and modelled via *has specified input/output* relationships.

#### Semantics of the multivariate linear mixed model

A semantic description of the multivariate LMM (as described in Supplementary File 1, model (2)) is an extension of the schema of the univariate mixed model (Fig. 4) by the inclusion of some additional concepts. Unlike the univariate model, where the relationship between a model parameter and a dependent variable is immediate, the relationship here is defined through the additional coefficients. For example, in the case of growth curve modelling, the coefficients frequently represent polynomial trends over time. For covariances of random effects, the concepts can be kept as in Fig. 4; however, here, the covariances also pertain to pairs of effects for different dimensions (or directly for different variables).

Technically, the evaluation of these models is usually done by encoding the multiple dependent variables as one continuous variable associated with a categorical classifying variable. In the semantic model, we make use of this pattern to maintain the consistency of the model component description as in the univariate model (the *term* is *about* a *variable*, and the *term effect* is *about* a part of the *variable*; hence, multiple *continuous variables* form *part of* a compound *dependent variable)*. However, we maintain the identity of separate dependent variables for the sake of accurate *dataset* and *effect* descriptions to avoid blurring the actual study design and to allow us to clearly identify the separate outputs.

#### Broader context of the model

**Fig. 7:**
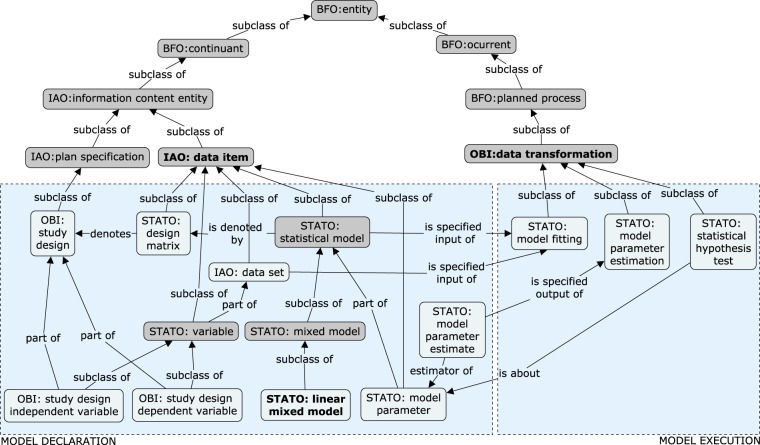
Terms related to the linear mixed model and inference embedded in the general schema of knowledge, defined through upper-level ontologies: BFO, IAO, and OBI. The generalisations of the classes used for the LMM entities so far are marked in dark grey.

Having described the knowledge schema directly related to the LMM, we can analyse the place of modelling from the wider perspective provided by the STATO and more abstract, underlying ontologies: Ontology for Biomedical Investigations (OBI), Information Artifact Ontology (IAO) and Basic Formal Ontology (BFO). Figure 7 illustrates a schema showing how the LMM concepts are linked to other terms.

An important IAO class is a *data item*, defined as an *information content entity* that is “intended to be a truthful statement about something”. Several terms in the graph are *data items*: *dataset*, defined as an “aggregate of *data items* that have something in common”, which in our case is the part of the *study design variables* containing the data points themselves; *linear mixed model*, being a subclass of a more general *statistical model*; *design matrix*; and *model parameter*. This shows that the considered ontologies attach to the term *data* a meaning much broader than the one restricted to experimental observations usually used in texts on statistical data analysis.

Another important OBI class is *data transformation*, a process that “produces output data from input data”; this class contains *model fitting* and its subprocesses, which have already been discussed.

The rest of the external classes shown in Fig. 7 define the place of the knowledge considered in this paper in the upper-level ontologies. The graph shows that at this level, both the *study design* and *statistical model* with its *parameters* are *continuants*, that is, entities that “persist, endure, or continue to exist through time while maintaining their identity”. On the other hand, all the *data transformations* used for inference are *planned processes*, or *occurrents*, that is, entities “that unfold themselves in time”. This important distinction between the two subsets of terms necessary for the semantic description of the LMM is eventually resolved by the highest BFO class *entity*, defined as “anything that exists or has existed or will exist”.

## Results

Application of the proposed semantic model to the LMM analysis of experimental data can be done by annotating individual elements of the analysis with the corresponding ontological concepts. All necessary terms (defined in either the extended STATO-LMM ontology, the core STATO ontology, or the underlying upper-level ontologies) are available through the STATO-LMM ontology module. As a result, the concepts are linked as in the presented semantic schema to form an RDF graph. This graph, serialised to a chosen format, can be uploaded to an RDF triple store and used in search and data discovery.

To facilitate the process of creating the RDF graphs, we provide an R package *semLMM*^33^ that allows us to automatically build a semantic model from the LMM analysis performed in the R statistical software (in the *lme4* and *nlme* packages).

### Exemplary analyses

Below, we briefly introduce exemplary LMM analyses of scientific datasets of different complexity to be modelled. Their detailed descriptions can be found in Supplementary File 1. The corresponding semantic models are given in Additional Examples^34^ and can also be explored as RDF triples or as visual graphs in an online database (a proof-of-concept GraphDB triple store) at http://lmm.cropnet.pl. The purpose of these examples is to illustrate basic use cases typically seen in the LMM analyses of experimental datasets and thus to demonstrate the scope of semantic models to be used later in Exemplary Queries for data search and discovery.

#### Example 1

Consider a simple dataset consisting of 6 observations obtained from an experiment performed for 3 different treatments in 2 blocks. The analysis is done with the linear mixed model approach, denoted informally as:

$$y=Treatment+Block+Error$$

The block effects are assumed to be random uncorrelated variables having the same variance; likewise, the error term. Estimates of the model parameters (the effects for each treatment, the block and error variances, and the contrasts between treatments) and their significance are sought. Together with the description of the methods applied, they constitute a summary of the dataset.

A graph presenting the basic elements of the semantic model of the LMM for this use case is shown in Supplementary File 3.

#### Example 2

Consider an extension of Example 1 to a situation where the observation is repeated at four time points. Data analysis by a multivariate linear mixed model can be denoted as:

$$y=Treatment.Time+Block.Time+Error$$

Block effects are assumed to be random and autoregressive in time; thus, the corresponding covariance structure is defined by two parameters (variance and autoregression coefficient). The error is assumed to be time-dependent and is described through a diagonal covariance structure depending on four parameters (the error variances for consecutive time points). The model parameters, i.e., 12 fixed effects for treatments in time (interpreted as marginal means) and six variance parameters, are estimated and tested as in Example 1, and they constitute a summary of the dataset analysis.

#### Example 3

Let us consider a more complicated example of a one-dimensional LMM based on an experiment that involves plant phenotyping (see http://cropnet.pl/plantphenodb/index.php?id=250). In this study, a number of plant genotypes were grown over two years in plots arranged in blocks. Among many traits, the heading date and grain yield were observed. The corresponding model to explain the grain yield can be summarised in informal notation as:

$$Yield=m+Genotype+Year+Genotype.\,Year+HeadingDate+Block+Error$$

where a general mean *m* and a simple covariance structure of the *Error* are assumed. The effects of the genotype, year, their interaction, and the covariate heading date are assumed fixed, while block effects are random, independent, and with a simple covariance structure; likewise, the error. Estimates of all the fixed effects and variance parameters, tests for the fixed effects, and the marginal means for all factor level combinations are needed to draw scientific conclusions from the experiment. A summary including the statistics and their provenance can be used for dataset documentation and search purposes.

### Exemplary queries

Below, we demonstrate the benefits of the structured LMM results for the exploration of datasets from scientific studies. The collection of semantic models, aggregating the results of statistical analyses of the experimental datasets, accommodates data summaries and documents the applied analytic approaches in a FAIR way. Ontological annotations of their individual elements enable the use of semantic reasoning in data processing. Asking adequate queries allows us to discover the studies that report specific statistical conclusions and to analyse the investigative methodology.

The use cases discussed below concern semantic models of statistical analyses of the results of agricultural scientific experiments, such as the Example 3 described above. In a general scenario for these experiments, a number of plants (representing different species and varieties) are cultivated under different environmental conditions (e.g., soil or weather attributes) and treated with different experimental factors (e.g., drought or chemicals), and a number of phenotypic traits are observed (e.g., plant height or yield). The corresponding datasets frequently undergo statistical analysis by the LMM (e.g.^35^) to explain the traits by means of other characteristics.

The use cases, formally specified as queries in the SPARQL language, are provided in Additional Examples^34^ and can be run in a triple store at http://lmm.cropnet.pl/sparql.

**Q1**: Find statistical models for a particular dataset

The same dataset can be analysed differently. Depending on the research type, an analyst can decide to use only one specific model or to try multiple modelling assumptions; one or more LMMs can be documented and stored. The query allows one to retrieve all statistical models used to analyse a particular dataset. The obtainment of the specification of each LMM (e.g., by formulas, terms, covariance structures, associated estimation and testing methods) enables researchers to study and repeat the same analysis (e.g., to validate the results for the original data, reanalyse an updated dataset or apply the same model to another observed variable). In the case of multiple LMMs per dataset, verification of the model fit criteria can be performed in subsequent steps of the analysis for statistical model selection (e.g., “Should all environmental attributes be used in further research?”). Thus, the idea behind this query is to simply document the analysis, and the usage of explicit semantics for its description can be beneficial in providing its unambiguous interpretation. http://lmm.cropnet.pl/sparql?savedQueryName=models.

**Q2**: List the dependent and/or independent variables

The coexistence of variables in experimental datasets can be indicative of the research directions undertaken in the collected studies. This knowledge of the available results is useful to identify reference studies or to conduct research accordingly, e.g., to replicate, enrich or bypass the existing approaches. A set of useful queries about the analysed variables allows one to carry out the following:

- check what variables were analysed together and in which studies (e.g., “Is there any experiment where both chemical X and chemical Y were used?”, “Were rainfall and plant height co-analysed somewhere?”) http://lmm.cropnet.pl/sparql?savedQueryName=variables.
- identify the studies that attempted to explain particular observed variables and to check what variables were considered explanatory for them, successfully or not (e.g., “In which studies was plant yield investigated, and which factors were applied in the experiment?”) http://lmm.cropnet.pl/sparql?savedQueryName=independent%20variables.
- identify the studies that report the significant role of a specific explanatory variable for explaining observed variables (“Which phenotypic traits significantly differ among plant varieties? Which studies report that?”, and “Are there studies that show evidence for the relevance of chemical X in explaining the plant height?”) http://lmm.cropnet.pl/sparql?savedQueryName=dependent%20variables.

**Q3**: Find the most important fixed effects

Certain values of the explanatory variables can specifically correlate with interesting behaviours of the observed individuals. Identification of the most extreme fixed effects (the biggest contrasts with the mean value of the observed variable) and their corresponding variable levels allows one to pay special attention to those particular values and the studies they appeared in (“Are there any specific plant varieties that produce extraordinarily big yields? Where was this observed?”). http://lmm.cropnet.pl/sparql?savedQueryName=significant%20fixed%20effects.

**Q4**: Find the largest variance components

Some explanatory variables, the individual values of which are not specifically interesting (being highly multivalued or outside of the experimenter’s control), are statistically modelled as random effects. In these cases, it is important to generally know how seriously they influence the observed variables. A query about the independent variables with the largest variances of random effects, i.e., those that contribute to the largest variability of the observed trait, allows one to identify potentially relevant experimental factors from existing studies (“For which environmental attributes is the yield most stable, and for which does it differentiate?”, “Is there any genotypic variability of the observed trait that is worth studying in further genotypic analyses, e.g., QTL detection?”). http://lmm.cropnet.pl/sparql?savedQueryName=variance%20components.

**Q5**: Find the marginal means of a specific dependent variable

In factorial experiments, where different sets of variables are investigated, it is crucial to determine how different combinations of the factors perform. A query that returns the marginal means of a specific trait for different levels of independent variables allows one to analyse the behaviour of the observed variable across all experimental groups and hence to identify the cases where especially interesting or favourable results were observed (e.g., “For which conditions were the highest plants observed?”). http://lmm.cropnet.pl/sparql?savedQueryName=marginal%20means.

## Discussion

In this work, we focused on providing explicit semantics for the elements of LMM analysis. The described methodology can be used in applications of the LMM to any dataset. The domain of scientific experimentation is a specifically justified use case to start with, as LMMs are frequently used in scientific studies where designed experiments are performed. However, specific cases of LMM analyses also include fixed effect models, linear regression models (e.g., linear regression fitted by least squares, or linear regression using shrinkage, such as LASSO regression for variable selection or ridge regression for multicollinearity) and random effect models. Thus, there is a vast area of applications of LMM analysis, and hence, our approach to documenting it can also be broadly applicable.

Depending on the needs, certain perspectives of the LMM analysis might be preferred in the semantic description, such as a static view of the estimates and statistics, a process-centred view of the fitting stages and methods, or a bare model declaration. In this paper, we concentrate on the former, where a semantically modelled LMM is considered as a proper summary of a specific experiment with the set of dependent and independent variables defined by the experimental design. There are, however, situations in which LMMs are used in the context of feature extraction and selection with the aim of providing a formulation optimal for prediction. In genomic selection (GS), a mixed model is sought that provides a good prediction of an organism’s phenotype from a set of independent variables describing its genotype at many loci^36,37^. In chemometrics and materials science, a multiple linear regression model is sought that describes the (quantitative) structure–activity relationship (QSAR) and provides good prediction of a compound’s activity on the basis of its physical or chemical properties^38^. In these applications, as well as frequently in the domain of observational studies, the number of available and potentially useful independent variables exceeds the number of observations, which prohibits the application of the classical estimation method. The attention is shifted from the individual model parameters and their statistical significance, which are not of primary interest, to the predictive value of the whole model and its application to subsequent datasets. For now, we do not apply the proposed approach in this context, but we claim that this extension is possible and can be pursued by interested teams in the appropriate domains of applications of linear model fitting.

The application of public ontologies rooted in the general knowledge schema allows the annotated statistical concepts to be placed in a general context. Hence, LMMs and other statistical approaches to data analysis, when documented with an STATO-based semantic model, can be semantically related to each other and processed analogically in the sense of semantic reasoning and technological aspects (RDF and SPARQL). Thus, for the sake of different statistical analyses and data integration, further specific modules extending the STATO can be developed.

In the description of the proposed annotation system, we did not elaborate on the methodological aspects of fitting linear mixed models related to methods of parameter estimation and hypothesis testing. This is a vast area of consideration in mathematical statistics, and we were able to mention only the most frequently used methods for estimation and basic statistical tests. The proposed annotation is - up to a certain point, in the layer of the model and its parameters - independent of the methodology applied to obtain the estimates and make decisions on the tested hypotheses. However, we realise that the various statistical approaches to linear mixed modelling can provide different results, and it is very important to understand the method actually used. Many concepts that cover these specific methodological aspects are provided by the STATO and can be used in specific implementations to extend annotations; some others, such as the concepts describing the modelling of heterogeneous error variances or multiple error terms (in, e.g., split-plot designs), the modelling of non-orthogonal data (the interpretation of model parameters or the order of hypothesis testing), or the use of the bilinear form of the multivariate LMM, need further work.

The issue of the statistical methodology is also relevant when considering the various software programs used to fit linear mixed models. Statistical packages such as R (*lme4*^39^ in Example 1; *nlme*^40^ in Example 3) or Genstat^41^ (Example 2) usually address the non-estimability of fixed effects in a similar way by providing estimates of a set of selected (linearly independent) parametric functions. In each case, it is important for the user to understand the exact meaning of the estimated effects, which depends on the design of the experiment. Both systems, R and Genstat, can provide the marginal means that are interpreted as absolute effects, which, to some extent, solves the problem of the interpretation of the partial results of the basic estimation. However, a comparison of the whole methodology used in LMM fitting with these and other packages is beyond the scope of this paper.

In our derivation, we restrict attention to an LMM with binary design matrices. With this assumption, the model parameters yield a standard interpretation of the effects and pairwise contrasts the interpretation of the relative effects. With other forms of the design matrices, the interpretation can become more complicated. Additionally, other types of linear parametric functions, with coefficients different than just −1, 0 and 1, can be useful. The semantic annotation of these models and functions is, of course, possible but requires more concepts, that is, extension of the model presented here. The same concerns a larger class of possibly nonlinear functions that can be defined only by proper formulae.

Domain-specific semantics of the datasets, which allow one to understand the underlying research questions, are beyond the scope of this paper; nonetheless, they are crucial for real-life use cases where the LMM is frequently applied. We assume that all inputs to the modelling, i.e., the dependent and independent variables, come from a properly annotated dataset constructed according to domain-specific standards (such as the Minimum Information recommendations, e.g., MIAPPE^42,43^), and this annotation provides domain-specific semantics. This information is necessary to properly construct the statistical model and interpret the obtained estimates in terms of the variable names, units, etc., and to exploit the ontological knowledge for experimental metadata-based semantic search (e.g.^44^) to further improve the data discoverability.

## Conclusions

In this work, we propose a semantic model of experimental data analysis with a linear mixed model approach. We summarise the underlying statistical theory behind the LMM analysis and its computational aspects and make use of existing terms from publicly available ontologies, mainly the STATO, to construct a semantic model of the LMM analysis and its results. Having identified some aspects of the LMM description not sufficiently covered by the existing terminologies, we enriched the STATO with the missing concepts.

We hope that the proposed semantic model of LMM analysis, by providing a comprehensive image of the process, can improve the general understanding of statistical modelling by researchers with different levels of expertise and background.

The modelling scope demonstrated in this paper is adjusted to the needs of experimental data management. The proposed semantic model is primarily supposed to explain the core assumption of the statistical modelling process and to provide a context for the statistical conclusions obtained from the analysis. Comprehensive dataset summaries contribute to a better understanding of the structure of the research data, while semantic annotation is essential for the interpretation of shared datasets. We hope that this work can contribute to the promotion of semantic approaches to research data description, especially the richer description of processed data to enhance their interoperability.

Although the present study describes a methodology related to formalising the description of linear mixed models for summarising experimental datasets, with the goal of efficient automated processing, the general approach of using a formal representation of the data analysis for scientific data discovery is also applicable to other data processing methods, in particular those that produce uniform, interpretable results. We are convinced that the publication of structured, semantically annotated results of experimental data analyses is necessary to allow efficient research data exploration. This will enable data discovery and stimulate data integration. Ideally, automatic processing and reasoning from scientific knowledge graphs can lead to the recognition of unknown facts. We hope that this work, by supporting open science, contributes to future progress in scientific research.

## Supplementary information

Supplementary File 1

Suplementary File 2

Supplementary File 3

## Data Availability

The data generated during the current study are available in the GitHub repository https://github.com/hcwi/SemanticLMM and Zenodo^33^. The datasets that served as use cases (i.e., raw experimental data from plant phenotyping experiments) were derived from the following public domain resources: PlantPhenoDB (http://cropnet.pl/plantphenodb) and URGI Plant and Fungi Dataverse (https://data.inra.fr), in particular the DROPS project data^35^.

## Code availability

The code that supports the application of the results of this study is available in the GitHub repository https://github.com/hcwi/SemanticLMM and Zenodo^33^.
