# Supplementary material for: Semantic concept schema of the linear mixed model of experimental observations
Source: Sci Data. 2020 Feb 27;7:70. doi: 10.1038/s41597-020-0409-7 (PMC7046786; doi:10.1038/s41597-020-0409-7)
Supplement: Supplementary file 1 [file 41597_2020_409_MOESM1_ESM.docx]

**Supplementary File 1.** **Mathematical description of the linear mixed model analysis.**

This document provides a theoretical background for the semantic model of the LMM proposed in the paper.

***Linear mixed modelling***

Below, an extended mathematical description of linear mixed models for univariate and multivariate case is given.

### Univariate (one-dimensional) model declaration

Let **y** denote a vector of observations of a random variable on *n* experimental units. We want to make inferences about unknown parameters of its distribution: its expectation E(**y**) = **µ** and its variance-covariance matrix Var(**y**) = **Σ_y_**. In general, **µ** is a vector of *n* possibly different elements and **Σ_y_** is a symmetric matrix of *n*(*n*+1)/2 possibly different elements. Modelling implies trying to discover whether the structure of **µ** or **Σ_y_** is in fact simpler, i.e., fewer different expectations and (co)variances can adequately describe the distribution of **y**.

This “simplification” utilizes “explanatory” variables to identify the parameters that can be considered not different, or equal to some constants. The variables explaining the structure of **µ** are said to exert "fixed effects" on **y**, while those describing the structure of **Σ_y_** exert "random effects" on **y**. The modelled variable is called a **dependent variable**, and the explanatory ones – **independent variables**. When relationships between the variables are assumed to be linear, the model is realised as a **linear mixed model**

**y = X**_1_**β**_1_ **+ X**_2_**β**_2_ **+ e** (1)

Here, the vector **β**_1_ consists of unknown constants called **fixed effects,** and the vector **β**_2_ consists of unobservable random variables called **random effects**. **β**_2_ is assumed to be normally distributed with expectation zero and variance-covariance matrix **Σ**. The vectors **β**_1_, **β**_2_ and the matrix **Σ** form a set of **model parameters**. Matrices **X**_1_ and **X**_2_, called **design matrices**, are known; they can be constructed from the observations of independent variables. The vector **e** consists of **random error effects** (independent of **β**_2_, normally distributed with expectation zero and covariance matrix **Σ**_e_) that represent the variability not otherwise accounted for in the model. In our derivation, for simplicity, we assume that different experimental units have homogeneous error variance and are independent: **Σ**_e_ **=** $\sigma_{\boldsymbol{e}}^{\boldsymbol{2}}$**I** (where **I** is an identity matrix). Hence, the **error variance**, $\sigma_{e}^{2},$ becomes another model parameter.

The notation used in (1) allows us to conveniently extend the formulae to a multivariate case of LMM, which we consider in subsequent sections. An equivalent, popular equation of the LMM is **y =** **Xβ** + **Zu** + **e**, where **β** denotes the vector of fixed effects, **u** − the vector of random effects, and **X** and **Z** are design matrices (see, e.g., ^1^ p.159, ^2, 3^).

Explanatory variables in designed experiments can be of two types: categorical or continuous. A **categorical variable** (called also a **factor**) takes values from a discrete set of **categories** (or **levels**) and describes a classification of experimental units into groups (e.g., according to biosource, batch, or treatment). It introduces the elements of vectors **β**_1_ or **β**_2_ that represent, respectively, fixed or random effects of individual categories. The columns of the design matrices **X**_1_ or **X**_2_ that correspond to these parameters are composed of zeros and ones which reflect the assignment of categories to experimental units. The second type of explanatory variables are observed **continuous variables,** which are used as **covariates** in the model. An effect of a continuous explanatory variable is usually represented by one element of **β**_1_ or **β**_2_ and is interpreted as a regression coefficient. The column of the design matrix **X**_1_ or **X**_2_ that corresponds to the regression parameter is composed of the observations (values) of the covariate.

The term “effect” is understood as a consequence of the application of a particular factor level on the distribution of the dependent variable. Effects can be exerted by explanatory variables independently of other explanatory variables or not. In the latter case, we talk about **interaction,** that is, a situation where the effects of one variable vary for different values of another variable; an interaction can concern a pair of categorical variables ("varying effect of categories of one variable for different categories of another variable") or a continuous and a categorical variable ("regression coefficient varying with categories of another variable"). An interaction can exist between independent variables with fixed effects, random effects or mixtures of both (“variances or covariances of some random effects vary with values of another variable”). Higher order interactions (for triples, quadruples, etc. of variables) are generalisations of the pairwise interaction. So-called nested effects of a variable are effects that can be exerted only in interaction with another variable which has an effect on its own.

To ease the description of models and guide their algebraic formulation, models can be declared by specifying their “parts” that address specific individual variables or their interactions. The parts are called **model terms**. Depending on the role of the underlying explanatory variables, the terms are fixed or random and they correspond, respectively, to submatrices of **X**_1_ or **X**_2_ which introduce to the model the parameters pertaining to those variables or their interaction, called “**term effects**”. A special fixed term that does not address any independent variable, called **intercept,** is used in some models; its representation in the design matrix is a vector of *n* ones and the corresponding model parameter stands for a **general mean**.

In the most general formulation of a random model term, all variances and covariances of its effects can be different. The corresponding covariance matrix is called unstructured and it brings in many parameters to the model. Thus, it is reasonable to search for some simplifications based on the *a priori* knowledge or preliminary data analysis that allow us to assume that the variances and covariances of the term effects can be defined through a function characterised by a smaller set of parameters, called **variance parameters**. Examples of such **covariance structures** are: scaled identity (sphericity), diagonal, autoregressive ^4, 5^.

### Statistical inference - model execution

Deducing the properties of probability distribution for the quantities of interest, i.e., those about which we wish to draw inference from the formulated model, comprises model parameter **estimation** and **hypothesis testing**.

Estimation consists in finding optimal (according to some statistical principle – such as unbiasedness or minimum variance) values of the model parameters, called **model** **parameter estimates**. Except for very simple models, more fixed parameters are used to formulate the model mathematically than can be estimated (due to collinearity of columns of design matrices). A workaround is to compute, instead of the estimates of **β**_1_, estimates of **parametric functions** of **β**_1_ which are of interest to the analyst and have unique estimates (so-called estimable functions, e.g., ^6^ p. 223). Simple and common examples of such parametric functions are differences between pairs of fixed effects, called pairwise **contrasts**, which represent **relative effects** of two categories (one versus another) or means of **y** for particular values of independent variables, called **predictions from the model** or **marginal means** (cf. ^1^). Parametric functions of variance parameters (such as ratios of appropriate variances) can also be estimated (e.g., in plant breeding, an important parametric function is called heritability).

For a proper presentation of experimental results, any set of estimates computed from the observed data should contain information about their precision (variability). This role is played by **standard errors** of “point” estimates (i.e., square roots of their **variances**) or **confidence intervals**, defined by their **confidence levels**, and the **lower** and **upper limits**.

The rules of obtaining estimates are expressed by mathematical formulae and are called parameter **estimators** (c.f. ^7^ def. 1.2, p. 4); as functions of **y**, they are random variables. The most frequently used estimators of fixed effects are empirical estimators based on Best Linear Unbiased Estimators (**BLUE**) ^8, 9, 10^. Optimal values of random effects can be found through a procedure called **prediction**, with the most frequent realisation in the form of Best Linear Unbiased Predictor (**BLUP**) ^8, 11^. We note that prediction can be also understood as a special case of estimation, namely, the estimation of expectation of random effects conditional on the observed value of **y** ^2^. The most popular methods of estimation of variance parameters are Maximum Likelihood (**ML**) or Restricted Maximum Likelihood (**REML**) ^1, 12, 13^. Finally, the estimates of parametric functions (e.g., marginal means or estimates of contrasts) are typically obtained by the application of these functions to the estimates of model parameters.

Hypothesis testing consists in verifying statements (**hypotheses**) concerning model parameters or parametric functions. A decision on (non-)rejection of a **null hypothesis** is based on a computed value of an appropriate **test statistic** (usually related to the analysis of variance: *F*-statistic or Wald statistic for general hypotheses about all term parameters or *t*-statistic for individual parameters), for which, based on a calculated **number of degrees of freedom**, a **p-value** is obtained. In cases where a large number of hypotheses forming "families" are tested (e.g., detailed hypotheses concerning all effects of one term), specific methods of correction for simultaneous testing are used to obtain "corrected p-values" (**q-values**), false discovery rates (**FDR**) or family-wise error rates (**FWER**). For random effects, the hypotheses that are usually tested concern variance parameters or their functions ^14, 15^. The random effects can also be tested using the REML likelihood ratio test (REMLRT ^16^) for nested models with the same fixed effects.

*Multivariate model declaration*

A multivariate case concerns a number of observed dependent variables, say *p*, modelled simultaneously. The multivariate model is advantageous when correlations between the observed variables are not negligible and must be taken into account in modelling. Typical situations in which the multivariate model is applied are those where the dependent variables pertain to distinct types of measurements on the same experimental unit (e.g., multi-trait models) or where they pertain to one type of measurement on the same experimental units at different points in some space, such as time (e.g., growth curve models for panel or longitudinal data). After ^17, 18^, we consider the multivariate LMM in the bilinear form

**Y = X**_1_**B**_1_**Z**_1_**+ X**_2_**B**_2_**Z**_2_**+ E** (2)

where **Y** is an (*n* × *p*) matrix of observations, **X**_i_ are known (*n* × *m****_i_***), *i* = 1,2, design matrices, **B**_i_ are (*m****_i_*** × *r_i_*), *r_i_* ≤ *p,* matrices of fixed and random model effects, and **Z**_i_ are (*r****_i_*** × *p*) dimension-reducing matrices for fixed and random effects, respectively. For **Z**_1_ and **Z**_2_ equal to identity matrix (i.e., with *r_i_* = *p*), model (2) is a straightforward extension of (1) and defines fixed and random effects for all dependent variables. Other forms of **Z**_i_ matrices transform the effects and introduce new dimensions that correspond to their linear combinations, which, assuming *r_i_* < *p*, reduces the number of parameters to estimate (e.g., in the case of growth curve modelling, the number of rows of **Z_1_** can be equal to the order of polynomial trend over time and the necessary coefficients form a Vandermonde matrix). **E** is a matrix of random errors uncorrelated with **B**_2_**.** To avoid a high number of parameters to estimate, the covariance matrix of **B**_2_ is usually simplified by imposing some structure that declares that only the pairs of random effects pertaining to different dimensions (dependent variables) can be correlated.

***Exemplary analyses***

Below, a mathematical description of the exemplary analyses of datasets is given. The raw data, analysis results and corresponding semantic models are provided in the paper as Additional Examples.

*Example 1*

Consider an exemplary dataset consisting of 6 observations of a variable **y** obtained from an experiment performed for three different **treatments (**T1, T2 and T3) and in two **blocks (**B1 and B2). The dataset is analysed with the linear mixed model approach, denoted informally as follows:

*y = Treatment + Block + Error*

According to the notation (1), an LMM with a fixed term for treatments and a random term for blocks takes the form:

| 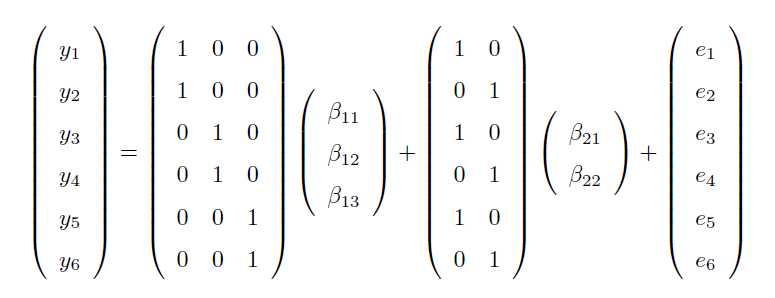 |
| --- |

where *β_ij_*, *i* = 1,2, j = 1,...,*m_i_*, denote the elements of vectors **β***_i_*. We assume that random block effects *β*_21_ and *β*_22_ are uncorrelated and have the same variance σ^2^ (that is, the random block term has a “scaled identity” covariance structure), similarly to the error, parametrised by$\sigma_{\boldsymbol{e}}^{\boldsymbol{2}}$ variance. Hence, our model parameters are {*β*_11_, *β*_12_, *β*_13_, *β*_21_, *β*_22_, $\sigma^{\boldsymbol{2}}$, $\sigma_{\boldsymbol{e}}^{\boldsymbol{2}}$}. In the model fitting process, we use REML method to estimate the variance parameters σ^2^ and σ_e_^2^. Then, estimates (empirical BLUEs) of the treatment effects (*β*_11_, *β*_12_, *β*_13_) are obtained (as functions of the estimates of the variance parameters) with their standard errors. In this simple model, the matrix **X**_1_ is of full rank, so all fixed effects can be estimated uniquely and their estimates are equal to marginal means for treatments (i.e., they constitute direct effects). A general hypothesis concerning no treatment effects (H_0_: *β*_11_ = *β*_12_ = *β*_13_) is tested by the Wald test, with computation of an *F*-statistic and a corresponding p-value. Additionally, contrasts between pairs of treatments – or their relative effects – with standard errors are also estimated, e.g., a contrast between T1 and T2 as a parametric function (*β*_12_ - *β*_11_).

*Example 2*

Consider an extension of Example 1 to a situation where the random variable **y** is observed at *p* = 4 time points. Data analysis by a multivariate linear mixed model can be denoted as:

*y = Treatment.Time + Block.Time + Error*

Without any reduction of dimensions for model effects (i.e., **Z***_i_* = **I**, *i* = 1,2, so we can skip it), this leads to the multivariate model (2) of the form


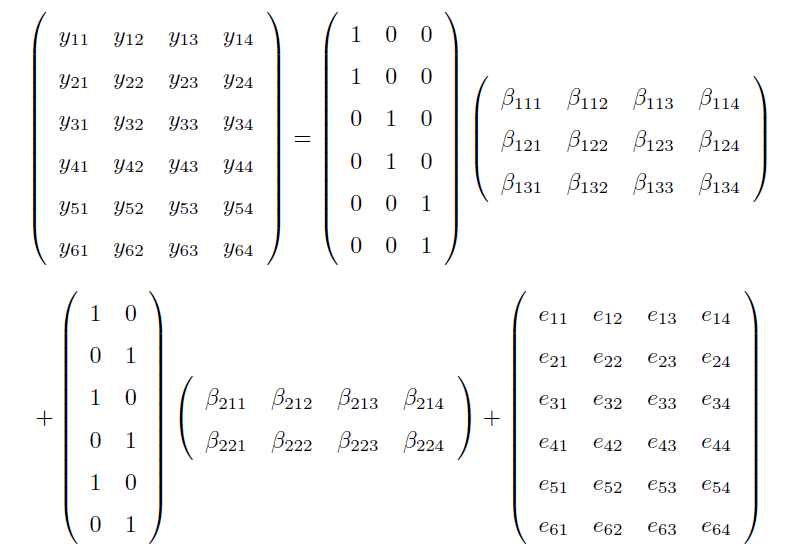


where *β_ijk_*, *i* = 1,2, *j* = 1,...,*m_i_*, *k* = 1,...,*p*, denote the elements of matrices **B***_i_*. We assume the covariance structure of random effects *β_2jk_* to be of the form **Σ** ⊗ **I**_2_, where **Σ** describes an autoregressive covariance structure of the first order, i.e., **Σ =** $\sigma^{\boldsymbol{2}}$**C**, where **C** is a symmetric matrix of order 4 with elements *c_ii_* = 1, *c_ij_* = *φ*c*_i_*_-1,_*_j_*; this covariance structure is defined by two parameters: $\sigma^{\boldsymbol{2}}$and *φ,* and ⊗ denotes a Kronecker product. We also assume that the error covariance structure is **Σ**_E_ ⊗ **I***_n_*, where **Σ**_E_ describes a diagonal covariance structure depending on four parameters that correspond to the error variances for consecutive time points. Model parameters, i.e., fixed effects *β_1jk_* interpreted as marginal means (no intercept in the model) and all variance parameters $\sigma^{\boldsymbol{2}}$**,** *φ* and $\sigma_{\boldsymbol{e}\boldsymbol{1}}^{\boldsymbol{2}}\boldsymbol{,}\sigma_{\boldsymbol{e}\boldsymbol{2}}^{\boldsymbol{2}}\boldsymbol{,}\sigma_{\boldsymbol{e}\boldsymbol{3}}^{\boldsymbol{2}}\boldsymbol{,}\sigma_{\boldsymbol{e}\boldsymbol{4}}^{\boldsymbol{2}}$, are estimated using the methods as in Example 1.

*Example 3*

Let us consider a more complicated example of data analysis by one-dimensional LMM, based on an experiment of plant phenotyping (see <http://cropnet.pl/plantphenodb/index.php?id=250>). In this study following a factorial experimental design, a number of plant genotypes (with fixed effects) were grown over two years (fixed effects) in plots arranged in blocks (random effects). Among many traits, heading date (a covariate) and grain yield (a modelled variable) were observed. A corresponding model can be summarised in an informal notation as:

*Yield = m + Genotype + Year + Genotype.Year + HeadingDate + Block + Error*,

where a general mean *m* and simple (scaled identity) covariance structures of *Block* and *Error* are assumed. Apart from quantifying all of the above-named effects (i.e., model parameters for all fixed effects and variances for random block effects, $\sigma^{\boldsymbol{2}}$, and for the error, $\sigma_{\boldsymbol{e}}^{\boldsymbol{2}}$), *F* tests and *t* tests for fixed effects are carried out and marginal means for factor level combinations are obtained from the analysis.

**References**

- - - 1. Searle, S. R., Casella, G. & McCulloch, C. E. *Variance Components*. (John Wiley & Sons, 2006).
      2. Gumedze, F. N. & Dunne, T. T. Parameter estimation and inference in the linear mixed model. *Linear Algebra Appl.* **435,** 1920–1944 (2011).
      3. Witkovský, V. Estimation, testing, and prediction regions of the fixed and random effects by solving the Henderson’s mixed model equations. *Meas. Sci. Rev.* **12,** (2012).
      4. Heck, R. H., Thomas, S. L. & Tabata, L. N. *Multilevel and Longitudinal Modeling with IBM SPSS*. (Taylor & Francis, 2010).
      5. Zimmerman, D. L. & Núñez-Antón, V. Parametric modelling of growth curve data: an overview. *Test* **10,** 1–73 (2001).
      6. Rao, C. R. *Linear Statistical Inference And Its Applications*. 625 (John Wiley & Sons, 1973).
      7. Lehmann, E. L. & Casella, G. *Theory of Point Estimation*. (Springer-Verlag, 1998). doi:10.1007/b98854
      8. Henderson, C. R. Best linear unbiased estimation and prediction under a selection model. *Biometrics* **31,** 423–447 (1975).
      9. Harville, D. A. Accounting for the estimation of variances and covariances in prediction under general linear model: an overview. *Tatra Mt. Math. Publ.* **39,** 1–15 (2008).
      10. Jiang, J. On unbiasedness of the empirical BLUE and BLUP. *Stat. Probab. Lett.* **41,** 19–24 (1999).
      11. Robinson, G. K. That BLUP is a good thing: the estimation of random effects. *Stat. Sci.* **6,** 15–32 (1991).
      12. Patterson, H. D. & Thompson, R. Recovery of inter-block information when block sizes are unequal. *Biometrika* **58,** 545–554 (1971).
      13. Christensen, R. in *Plane Answers to Complex Questions* 291–331 (Springer, 2011).
      14. Khuri, A. I., Mathew, T. & Sinha, B. K. *Statistical Tests For Mixed Linear Models*. 384 (John Wiley & Sons, 1998).
      15. Michalski, A. & Zmyślony, R. Testing hypotheses for variance components in mixed linear models. *Statistics* **27,** 297–310 (1996).
      16. Self, S. G. & Liang, K.-Y. Asymptotic properties of maximum likelihood estimators and likelihood ratio tests under nonstandard conditions. *J. Am. Stat. Assoc.* **82,** 605–610 (1987).
      17. Žežula, I. Variance components estimability in the extended growth curve model. *Tatra Mt. Math. Publ.* **26,** 195–199 (2003).
      18. Žežula, I. Special variance structures in the growth curve model. *J. Multivar. Anal.* **97,** 606–618 (2006).
