# Supplementary material for: Semantic concept schema of the linear mixed model of experimental observations
Source: Sci Data. 2020 Feb 27;7:70. doi: 10.1038/s41597-020-0409-7 (PMC7046786; doi:10.1038/s41597-020-0409-7)
Supplement: Supplementary file 3 [file 41597_2020_409_MOESM3_ESM.pdf]

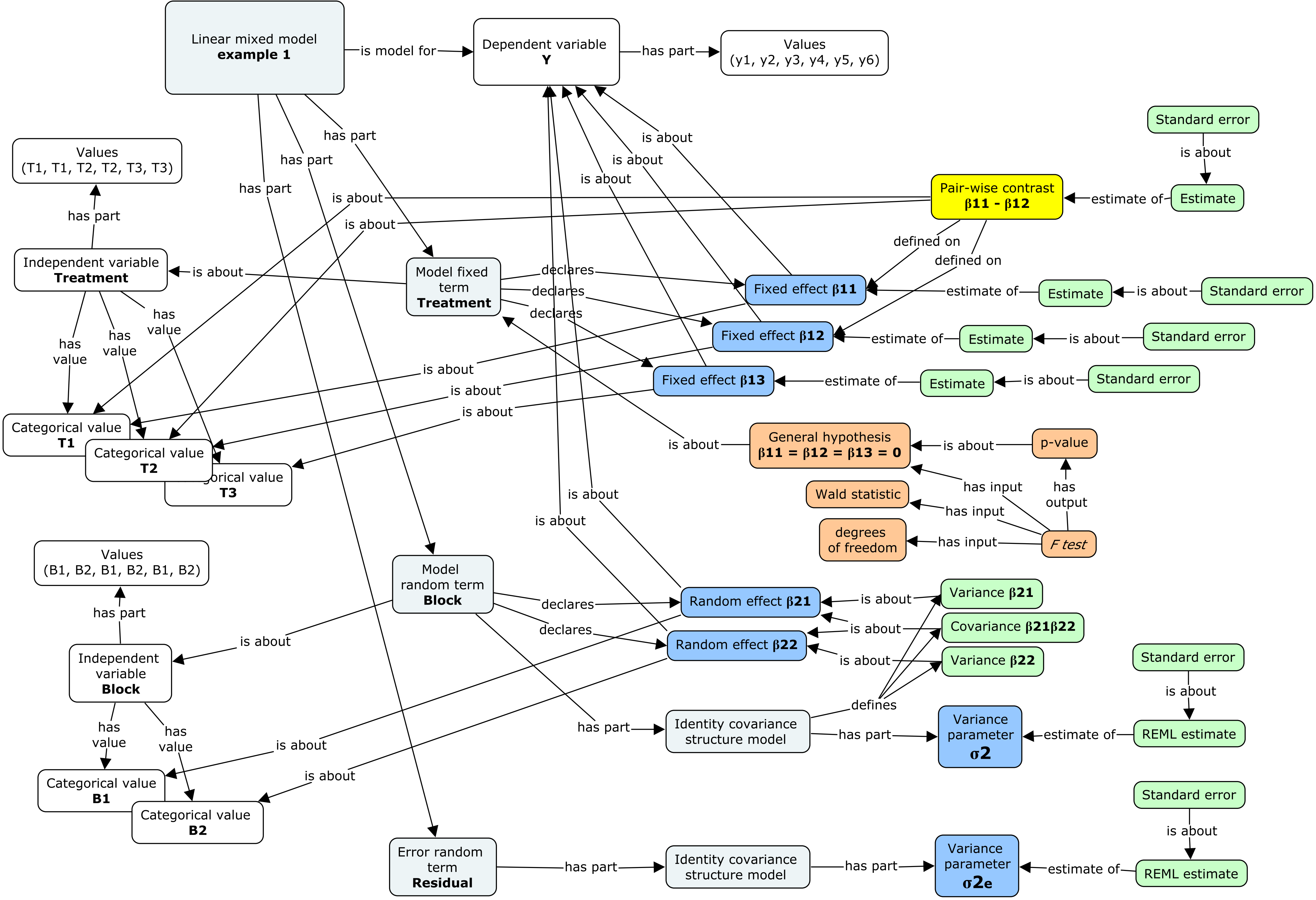

Supplementary File 3. **Graphical representation of the semantic model of Example 1**. A simplified graph illustrating the core elements in the semantic model of LMM analysis of a two–factor experiment. The dataset comprises 6 observations {y1 … y6} classifed at 3 levels {T1, T2, T3} of Treatment factor and 2 levels {B1, B2} of Block factor. Statistical analysis comprises estimating and testing of model fixed effects {β11, β12, β13} and estimating model random effects {β21, β22} and variance parameters {σ2, σ2e}. The nodes represent instances of the basic classes involved in statistical model declaration. Colours: white – variables and their values, blue – model parameters , yellow – parametric functions, green – estimation components, orange – testing components.
